# Assisted suicide in Germany: a survey on requests for and current practices of assisted suicide

**DOI:** 10.1186/s12910-026-01406-6

**Published:** 2026-02-13

**Authors:** Sabine Sommerlatte, E. Droese, C. Bausewein, T. Pollmächer, G. Marckmann, A. Simon, J. Schildmann

**Affiliations:** 1 Institute for History and Ethics of Medicine, Interdisciplinary Centre of Health Sciences, Martin Luther University of Halle-Wittenberg, Halle (Saale), Germany; 2 Department of Palliative Medicine, LMU University Hospital, Munich, Germany; 3 Donau-Altmühl-Kliniken, Ingolstadt, Department of Psychiatry and Psychotherapy, LMU University Hospital, Ludwig-Maximilians-University Munich, Munich, Germany; 4 Institute of Ethics, History and Theory of Medicine, LMU Munich, Munich, Germany; 5 Academy of Ethics in Medicine, Department of Medical Ethics and History of Medicine, University Medical Center Göttingen, Göttingen, Germany

**Keywords:** Assisted suicide, Standards for practice, Decisional capacity

## Abstract

**Background:**

Following the constitutional court ruling in 2020, which legalized assistance in suicide in Germany under certain conditions, the practice of assisted suicide in Germany has been developing rather dynamically regarding the number of assisted suicides as well as the number of institutions offering suicide assistance. Challenges include a competent assessment of decisional capacity and the choice of adequate drugs. The aim of this study was to provide information about characteristics of people requesting assistance with suicide and those who died by assisted suicide as well as information about the current practice of assisted suicide in Germany.

**Methods:**

We conducted an anonymous, open online survey with a convenience sample of 672 people who signed up for a webinar on professional standards of suicide assistance in April 2024. Descriptive statistical analysis of absolute and relative frequencies of the responses was performed. The results are provided as total numbers and percentages for either the whole sample (including those missing) or the subgroups analyzed. The free-text answers were coded independently by two researchers by means of qualitative content analysis.

**Results:**

A total of 128 of 234 participants who accessed the online survey were included. Key findings are, firstly, a small subgroup receiving a substantial number of requests and assisting suicide in a high number of cases. Secondly, a wide range of professional groups and nonprofessionals were confronted with requests, and thirdly, a great heterogeneity regarding the practice of suicide assistance was observed, i.e. regarding the drugs applied, the process of information and counselling, and the assessment of decisional capacity.

**Conclusions:**

Our data indicate a lack of standardization in assisted suicide practice in Germany, which is associated with certain risks such as complications for those seeking suicide assistance due to inappropriate medication, inequalities in access to suicide assistance and legal uncertainty for those providing suicide assistance. Standardizing assisted suicide practice could facilitate quality and legal certainty. Further research based on prospective registries can support transparency and standardization and contribute to an accountable practice of assisted suicide in the future.

**Supplementary Information:**

The online version contains supplementary material available at 10.1186/s12910-026-01406-6.

## Introduction

Before the ruling of the German Federal Constitutional Court in 2020, services for assistance in suicide (AS) were punishable under paragraph 217 of the German Criminal Code. As a result of several constitutional complaints filed by several parties, the German Federal Constitutional Court declared the criminalization of AS services unconstitutional on February 26, 2020 [1]. Consequently, AS is legal in Germany, similar to several other countries [2]. However, the practice and legal requirements of those countries allowing AS differ considerably [3]. In Canada for example, next to the decision-making capacity of the person requesting AS, additional eligibility criteria must be met, such as the presence of a grievous and irremediable medical condition [4]. In contrast, being (terminally) ill is a legal requirement for legally authorized AS neither in Switzerland nor in Germany [1, 5]. Regarding the practice of AS, in Oregon (USA) for example, lethal drugs are prescribed to eligible patients, who then may ingest the substance at a place and time of their own choice [6]. In Switzerland, AS often takes place at a location and time agreed between the person intending to die by AS and representatives of organizations for AS [7].

In Germany, the constitutional court defined “*Freiverantwortlichkeit*,” which encompasses decisional capacity, informed decision-making, voluntariness and stability of the wish to die, as the decisive eligibility criterion for lawful AS [8]. According to this judgement, “the general right of personality (Art. 2(1) in conjunction with Art. 1(1) of the Basic Law, Grundgesetz – GG) encompasses a right to a self-determined death. This right includes the freedom to take one’s own life and, as the case may be, resort to assistance provided voluntarily by third parties for this purpose” [1]. However, currently there is no explicit legal framework for AS in Germany that regulates, for example, the assessment of “Freiverantwortlichkeit”. Previous attempts to pass laws failed and the debate over whether further legal regulations are necessary is controversial [9]. While some consider legal regulations necessary to prevent abuse and increase transparency, others consider existing criminal law to be sufficient and see further legal regulations as a threat to autonomy and self-determination [10, 11].

Regardless of the absence of a legal framework, following the constitutional court ruling in 2020, the practice of AS in Germany has been developing rather dynamically regarding the number of AS as well as the increasing number of institutions offering AS [12]. However, little is known about the details regarding requests for AS and the current practice in Germany. In a recent series of articles assessing the practice of AS in the city of Munich based on death certificates, autopsy death certificates and reports, and toxicological reports, the authors suggest a number of challenges with current practice such as the assessment of decisional capacity and the choice of adequate drugs [13–16].

While there have been a number of publications internationally describing the characteristics of people who died by AS, there are less data available reporting the characteristics of those who request AS [17–21]. Details about both groups seem relevant to be able to inform the developing practice of dealing with requests as well as assisting in suicide. Against this background, the aims of this study were

1. To provide information about the characteristics of people requesting AS in Germany.
2. To provide information about the current practice of AS and the people who died by AS.

## Methods

### Study design and participants

We conducted an anonymous, open online survey with a convenience sample of 672 people who signed up for a webinar on professional standards of AS on April 12, 2024. The study was conducted via the Lime-Survey platform, which is located on the server of Martin Luther University Halle-Wittenberg. Emails with a survey link as well as a description of the study and its aims were sent to all applicants on March 28, 2024, followed by a reminder on April 12, 2024. People receiving the emails were invited to forward the survey to others possibly interested in the topics. The survey was closed on April 17, 2024.

### Questionnaire

The questionnaire was developed based on previous studies and piloted by the authors, further researchers with backgrounds in medicine, ethics and other disciplines, as well as research students at the institution of the first author [22–24]. It included multiple-choice questions along with options for free-text comments. Demographic information, personal experiences and information on the practice of AS was collected. Furthermore, participants were asked about the frequency of requests for AS during the last 12 months („Bitte geben Sie an, wie häufig Sie bezüglich Assistenz bei der Selbsttötung in den letzten 12 Monaten angefragt wurden.“/„Please indicate how often you have been asked to assist in suicide in the last 12 months.“). In addition, participants were asked to provide more detailed information about the person who last asked them about AS and the subsequent course of events, regardless of the time period. Therefore, individuals who stated that they had not received any requests in the last 12 months were also included in the study, provided that they were still able to report a case that occurred more than 12 months ago. The questionnaire with the questions included in this study are provided as an additional file [see Additional file 1].

### Data analysis & statistics

Descriptive statistical analysis of absolute and relative frequencies of responses was performed with IBM SPSS statistics version 28.0 for windows. The results are provided as total numbers and percentages for either the whole sample (including those missing) or the subgroups analyzed.

Free-text answers were analyzed by means of qualitative content analysis according to Kuckartz [25]. This concerns the questions regarding frequencies of requests for AS and actual AS provided, information and counseling, assessment of decisional capacity, the practice of AS and the time to death [see also Additional file 1]. First, SaS inductively created categories for coding the responses. Then, two researchers independently coded the responses using these categories and then compared and discussed the results (SaS, ED). Discrepancies were discussed with JS. Since the qualitative data are derived solely from brief and partly bullet-pointed free-text responses, the categories are reported in absolute frequencies due to a lack of depth in the data.

## Results

### Sample characteristics and requests

A total of 128 of 234 participants who accessed the online survey were included. One duplicate was removed and 105 datasets were excluded due to lack of sufficient data for analysis (5 or less items completed). Sociodemographic characteristics of the participants are summarized in Table 1.

**Table 1:** Sample characteristics

	Participants	
	*n*	%
Gender		
Female	81	63.3
Male	45	35.2
Diverse	0	0.0
Missing	2	1.6
Age (years)		
18–30	1	0.8
31–40	10	7.8
41–50	21	16.4
51–60	41	32.0
61–70	31	24.2
71–80	17	13.3
81–90	5	3.9
91–100	2	1.6
Missing	0	0.0
Profession/role (multiple answers possible)		
Physician	51	39.8
Psychologist/psychotherapist	17	13.3
Nursing professional	16	12.5
Pastoral caregiver	11	8.6
Social worker	9	7.0
Friend/acquaintance	9	7.0
Relative	7	5.5
Volunteer (e.g. hospice association/visiting service)	7	5.5
Physical therapist/occupational therapist/speech therapist	2	1.6
Lawyer	1	0.8

**Fig. 1:**
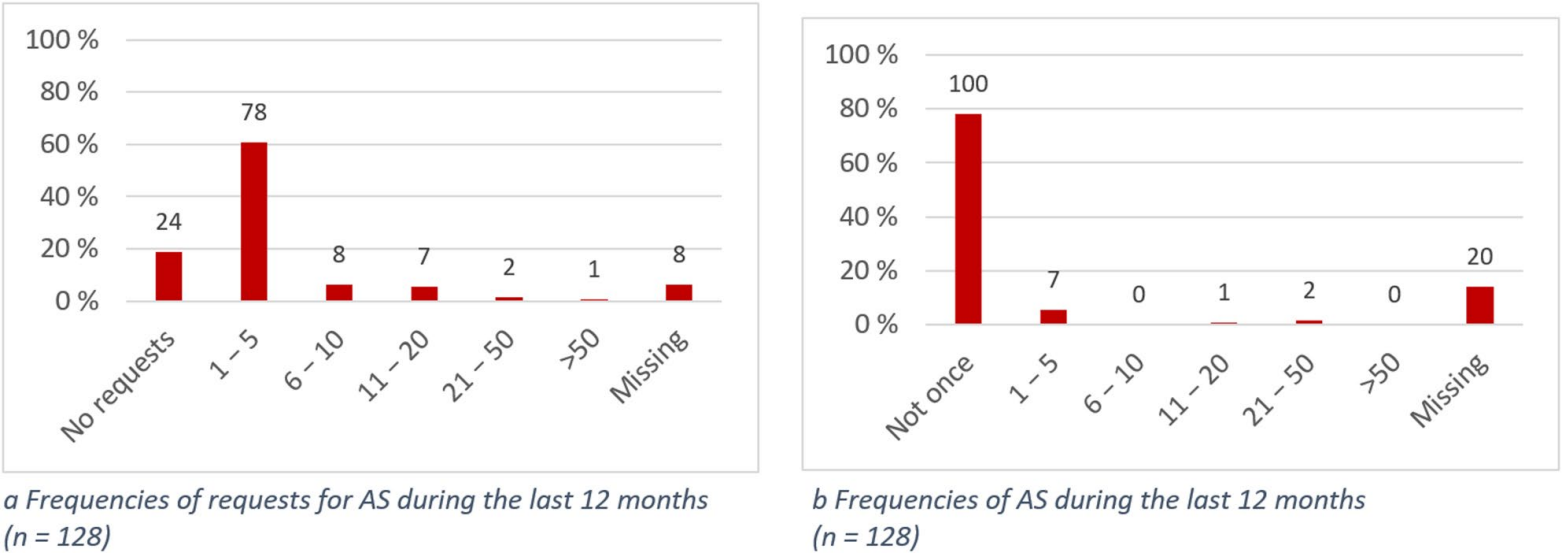
**a** Frequencies of requests for AS during the last 12 months (n = 128). **b** Frequencies of AS during the last 12 months (n = 128)

A total of 78/128 participants (60.9%) reported 1 to 5 requests for AS during the last 12 months; more than 10 requests were reported by 10/128 participants (7.8%); 24/128 participants (18.8%) reported no requests. Ten (7.8%) people stated that they had assisted with suicide in the last 12 months. Four of them were physicians, one person was a volunteer (e.g. hospice service or visiting service), one was a volunteer suicide assistant, one was a pastoral caregiver, one was a social worker, one was a physical therapist/occupational therapist/speech therapist and one person did not specify their occupation, but stated that they were involved in the case as a private individual. The frequency of reported AS was between 1 and 44 cases. Details are shown in Fig. 1a and b.

### People reported to have requested AS

In the following, we report information provided by participants on the last person who asked them for AS, including a subpopulation of 11 requesters who were reported to have eventually died by AS. The cases reported here are not limited to the last 12 months, but refer to the last person reported to have asked for AS, regardless of the time period. The results are, therefore, reported in relation to the complete sample (*n* = 128), even though some of the respondents (*n* = 24) stated that they had not received a request for AS in the last 12 months. AS was defined in the questionnaire as providing help to end one’s life, for example by prescribing or providing a lethal substance. The study participants reporting the cases were asked to provide more detailed information about the person who last asked them about AS and the subsequent course of events. They are therefore not necessarily identical to those who actually assisted in the respective suicide according to the above definition.

A wide age range was represented among the requesters, with the majority (94/128, 73.4%) being over 50 years old. The requesters had between none and four underlying illnesses, with a median number of 1 (IQR = 2). The most frequent diagnoses reported were cancer (35/128; 27.3%), neurological (25/128; 19.5%), and mental/psychiatric disorders (21/128; 16.4%). Only 8/128 (6.3%) had no diagnosed illness. Altogether, 37/128 requesters (28.9%) were cared for by a palliative care/hospice team. Just under half of the participants (59/128; 46.1%) stated that they would not have been surprised if the requester had died by natural causes within the next 6 months, 29/128 (22.7%) would have been surprised, and 10/128 (7.8%) stated that they did not know.

The level of education varied from having no school leaving certificate (1/128; 0.8%) to holding a university degree (45/128; 35.2%). A total of 54/128 (42.2%) of requesters were living in private homes at the time of the request. Altogether, 80/128 (62.5%) were reported to get by financially or live comfortably while 16/128 (12.5%) were reported to be able to get by only with (great) difficulty regarding their financial situation. Table 2 summarizes details of the sociodemographic and health-related aspects of the requesters as well as the subgroup of those people who were reported to have died by AS.

**Table 2:** Characteristics of the people reported to have requested AS (*n* = 128) and those reported to have died by AS (*n* = 11)

	People reported to have requested AS		Subpopulation of people reported to have died by AS
	*n*	%	*n*
Gender			
Female	52	40.6	4
Male	55	43.0	7
Diverse	1	0.8	0
Missing	20	15.6	0
Age (years)			
18–30	1	0.8	0
31–40	3	2.3	0
41–50	9	7.0	0
51–60	14	10.9	1
61–70	25	19.5	2
71–80	22	17.2	2
81–90	21	16.4	4
91–100	12	9.4	2
Missing	21	16.4	0
Level of education			
Secondary school^a^	5	3.9	0
Intermediate school^b^	20	15.6	2
Grammar school^c^	48	37.5	8
No school-leaving certificate	1	0.8	0
Unknown	30	23.4	1
Missing	24	18.8	0
Training/education			
Apprenticeship	26	20.3	2
University	45	35.2	7
Other education	3	2.3	1
Unknown	30	23.4	1
Missing	24	18.8	0
Marital status/children			
Single	33	25.8	5
Married/living in a partnership	41	32.0	6
Widowed	22	17.2	2
Children	32	25.0	4
Unknown	5	3.9	0
Missing	12	9.4	0
Financial situation			
Could live comfortably	53	41.4	9
Was able to get by	27	21.1	1
Had difficulty getting by	6	4.7	0
Had great difficulty getting by	10	7.8	1
Unknown	8	6.3	0
Missing	24	18.8	0
Diagnoses			
Cancer	35	27.3	3
Neurological disease	25	19.5	4
Mental/psychiatric disorder	21	16.4	0
Respiratory disease	10	7.8	1
Cardiovascular disease	9	7.0	1
Musculoskeletal disease	9	7.0	3
Endocrine disorder	3	2.3	0
Gastrointestinal disease	3	2.3	0
Metabolic disease	2	1.6	0
Infectious diseases	1	0.8	0
Other illness	17	13.3	3
No diagnosed illness	8	6.3	1
Unknown	3	2.3	0
Missing	18	14.1	0
Palliative care involvement			
Yes	37	28.9	4
No	60	46.9	7
Unknown	2	1.6	0
Missing	29	22.7	0
Place of residence			
Private home	54	42.2	7
Care facility	14	10.9	4
Hospital	12	9.4	0
Palliative care unit	3	2.3	0
Hospice	2	1.6	0
Other place	5	3.9	0
Unknown	2	1.6	0
Missing	36	28.1	0
Participant would have been surprised if the requester had died by natural causes within the next 6 months.			
Yes	29	22.7	2
No	59	46.1	7
Unknown	10	7.8	2
Missing	30	23.4	0

^a^ A secondary school in Germany (“Hauptschule”/”Mittelschule”) is a type of school that provides basic general education and is usually completed after grade 9 or 10. It places particular emphasis on practical teaching and preparation for vocational training. ^b^ An intermediate school (“Realschule”) provides an extended general education and is usually completed after grade 10. It is between the “Hauptschule” and “Gymnasium” in terms of academic level and prepares students for dual training programs, vocational schools, or the transition to the upper level of the “Gymnasium”. ^c^ A grammar school (“Gymnasium”) provides in-depth, science-oriented general education and is usually completed after twelve or thirteen years of schooling. It prepares students specifically for university studies, but also for demanding vocational training programs

A total of 52/128 participants (40.6%) reported that no AS was carried out, 9/128 (7.0%) stated that they had no further information about the case. Altogether, 15/128 (11.7%) reported that AS was carried out. Of these, 11 respondents (8.6%) provided details, which we report in the following. The characteristics of the subpopulation reported to have died by AS are also provided in Table 2.

Seven out of a total of eleven people who were reported to have died by AS were male. All of them were over 50 years old. Five of a total of eleven people were single. Ten out of a total of eleven people were able to live comfortably or get by financially, and only one person was able to get by with great difficulty. Between none and two underlying diseases were known. Three out of a total of eleven people were known to have had advanced cancer, and epileptic seizures caused by brain metastases were described in one of them. Three other people were known to have had a neurological disease, one with amyotrophic lateral sclerosis, one with cerebellar ataxia and one with polyneuropathy. One person had no known illness, but old age was specified as “other illness” in the free-text comment. The person was between 81 and 90 years old. None of the people were known to have had a mental illness.

Four out of a total of eleven people were cared for by a palliative care/hospice team. Two out of a total of eleven participants would have been surprised if the person had died of natural causes within the next 6 months, seven out of a total of eleven participants would not have been surprised and two out of a total of eleven did not know.

The time interval between the first request and the AS varied between four weeks and 24 months. The AS took place in private homes in 6/11 cases, in a care facility in 4/11 cases and in the mourning room of a funeral service in one case.

Friends or acquaintances were present in 10/11 cases, a doctor in 5/11 cases, relatives in 2/11 cases, and a nurse in one case.

Barbiturates/narcotics were used in 6/11 cases, in four of which thiopental was specifically stated as the substance. Helium was used in one case. Three people stated the form of administration, namely, oral (*n* = 1) and infusion (*n* = 2), and the substance applied was not known in one case.

The reported time between self-administration of a lethal substance and death ranged from five minutes to seven hours.

All eleven people who were reported to have died by AS were reported to act voluntarily, have decisional capacity and be informed about their situation and the options for action available.

Decisional capacity was assessed in 9/11 cases with involvement of physicians. Decisional capacity was assessed by more than one person in 6/11 cases. Lawyers and relatives were involved in 2/11 cases.

Eleven participants provided information about the person who informed the requester about options for action in the free-text comments. More than one person was involved in counseling in 8/11 cases. Physicians (*n* = 8), lawyers (*n* = 2), the family of the person who died by AS (*n* = 1), a nutritionist (*n* = 1), organizations which assist in suicide (*n* = 6), a nurse (*n* = 1), the nursing home (*n* = 1), a palliative care service (*n* = 1) and volunteers (*n* = 2) were mentioned regarding the provision of information prior to AS. The physician consulting and assisting the suicide was the same in one case. Four participants also provided details about the content of the consultation. These were alternative therapies and care options (*n* = 2), palliative treatment options (*n* = 2) and information about (forms of) AS (*n* = 2).

## Discussion

To the best of our knowledge, this is the largest study in Germany with details on requesters and people who were reported to have died by AS as well as details about practices of AS subsequent to the change in the legal situation in 2020.

Key findings are, firstly, a small subgroup receiving a substantial number of requests and assisting suicide in a high number of cases. Secondly, a wide range of professional groups and nonprofessionals were confronted with requests. Thirdly, self-reported data indicate a great heterogeneity regarding the practice of suicide assistance, i.e. regarding the drugs applied, the process of information and counselling, as well as the assessment of decisional capacity. This heterogeneity points to a lack of standardization of key elements of assisted suicide practice.

### Frequencies of requests

The majority of the 128 participants had received at least one request for AS in the last 12 months. A smaller subgroup of 10 respondents (7.9%) stated that they had actually assisted with a suicide in the last 12 months. A few respondents received a high number of requests and/or assisted with suicide more than 20 times. However, our data does not reveal who assisted in the suicide, but only who received the request. The “German Society for Humane Dying“ (Deutsche Gesellschaft für Humanes Sterben”/DGHS) states that it received 802 requests for AS in 2023 and assisted in 419 suicides [12, 26]. Table 3 shows an overview of the number of suicides assisted by three large German aid-in-dying organizations for the years 2020–2024.

**Table 3:** Number of suicides assisted by three large German aid-in-dying organizations for the years 2020–2024

	Number of assisted suicides (Germany)		
Year	DGHS	Dignitas Germany	Verein Sterbehilfe
2020	18	Unknown	103
2021	120	97	129
2022	229	199	139
2023	419	258	196
2024	623	183	171

Information provided by the German Society for Humane Dying (DGHS) [12, 26]

The fact that the study participants included representatives from a wide range of different groups of people and professions shows that the question of how to deal appropriately with requests for AS is of great relevance to a wide range of professions as well as nonprofessionals. Against this background, the question arises which competencies are needed to deal appropriately with requests for AS. The relatively high overall age of our sample was noticeable with 24/128 (18.8%) being over 70 years old. One explanation could be that people who are older are more interested in AS or that they are asked more frequently for assistance with suicide. The question arises as to whether a certain age or level of experience should be assumed for people who provide assistance with suicide.

### Characteristics of requesters

Overall, the group of requesters appears to be more diverse in terms of age, level of education and existing diagnoses compared to the subgroup reported to have died by AS. What was unusual was the relatively high proportion of men (7/11) compared to other studies [13, 18]. However, this interpretation is limited due to the small group size of eleven people who were reported to have died by AS.

Notably, some of the requesters and also one person who was reported to have eventually died by AS were reported to be in a difficult financial situation. There is concern that vulnerable people, for example, those of low socioeconomic status, could be pressured into AS [27]. However, data from Canada indicate that, while a large proportion of requests for medical aid in dying come from people of low socioeconomic status, the ratio of low to high socioeconomic status among those actually receiving medical aid in dying is balanced or even skewed toward those of a higher socioeconomic status [28–30]. German data from Gleich et al. [31] based on death certificates, autopsy death certificates and reports, and toxicological reports showed that 19/77 people, who died by AS in the city of Munich between 2020 and 2023, were academics. Whether and to what extent socioeconomic status plays a role in the requests for and access to assistance with suicide in Germany should be the subject of further investigation.

The most frequent diagnoses reported in both groups were cancer and neurological diseases, which is in line with previous studies [15, 18, 32]. Only a small proportion of the requesters, as well as one person who was reported to have died by AS, had no known preexisting disease.

A difference could be seen regarding psychiatric disorders, which were commonly reported among the requesters, whereas none of the people who were reported to have died by AS had a psychiatric diagnosis. In contrast to this finding, Schäffer et al. [16], who analyzed death certificates, identified diagnoses of depression, addiction and cognitive impairment/dementia in 26/68 patients who died by AS. This contradiction could be explained by a reporting bias in our data, as suicide assistance in patients with psychiatric disorders is controversial [33, 34]. In the context of AS, patients with psychiatric illnesses are often considered to be particularly complex and vulnerable, as these illnesses can potentially impair their decisional capacity [16, 35, 36]. However, the presence of a mental disorder must not automatically be interpreted as a lack of decisional capacity [36, 37].

### Practice of AS

Regarding information and counseling and the assessment of decisional capacity, the question arises as to who can provide such counseling and assess decisional capacity and what information the requesters need in order to be able to make an informed and autonomous decision.

In a survey with 745 members of the German Society for Hematology and Medical Oncology (DGHO), the majority of respondents (57.6%; *n* = 429) were in favor of a mandatory formal assessment of decisional capacity [3]. The disciplines most frequently mentioned as those that should be involved in counseling were professionals of palliative care (*n* = 617; 88.1%) and the discipline specialized in the disease of the patient [24].

In our survey, both the number of people providing information and counseling and assessing decisional capacity varied, as did their professional expertise and the content of the information provided. In most cases, physicians were involved in counseling (8/11) and the assessment of decisional capacity (9/11). It was reported that information was provided on alternative and palliative treatment options only in 2/11 cases. This is particularly remarkable, as 7/11 respondents stated that they would not have been surprised if the requester had died of natural causes within the next 6 months. Although our findings are based on self-reported data, and it is therefore not possible to make statements about the actual relevance of possible alternative or palliative treatment options in each case, we would argue that there are always alternatives to AS that should at least be communicated. The question is, of course, whether these options are “relevant” options from the requesters’ point of view.

In at least one case each it was reported that the physician consulting/assessing decisional capacity and assisting with the suicide was the same. Having different people conduct the assessment of decisional capacity and assist with the suicide can increase the objectivity and neutrality of the assessment and help to avoid potential conflicts of interest (e.g. financial or personal). For this reason, the assessment of eligibility criteria by at least two independent practitioners is mandatory according to some international practice standards, like in Canada and the Netherlands [35, 38]. Our data, therefore, indicate a possible bias regarding the provision of information and counseling, and the assessment of decisional capacity. This finding is substantiated by a German study from Schäffer et al. based on death certificates and further legal documents, which indicates that assessment of decisional capacity is often insufficient [16].

Regarding the procedures of AS, different substances and forms of administration (oral/intravenous) were reported. Gleich et al. [13] point out in their study the dangers of a lack of standardization in medication for AS because some medications used in AS practice are associated with a high risk of complications and delayed death. The authors therefore advocate the creation of a legal basis for the use of Pentobarbital, which is considered effective and safe, for the purpose of AS in Germany [13]. A safe and reliable practice with suitable medication and dosage forms is of the utmost importance to prevent potential harm to those affected.

## Conclusions

The lack of standardization in AS practice in Germany, which is indicated by our data, is associated with the risk of ethical, legal, and social problems. These include complications for those seeking AS due to inappropriate (use of) substances for AS. Furthermore, different standards for assessing eligibility criteria such as decisional capacity may lead to inequalities in access to AS and legal uncertainty for those (professions) assisting in suicide. The latter is particularly relevant given the lack of a legal framework in Germany, as demonstrated by recent convictions of individuals who have assisted in suicides [39]. Standardizing central elements of AS practice could therefore contribute to better quality and facilitate legal certainty and transparency. To achieve standardization, a meaningful database and transparent documentation of AS practice are necessary, since practices from other countries may only be applicable to Germany to a limited extent due to different legal or social standards. Our study provides an important basis for a starting point, but only allows for limited generalizability due to the selectivity of the sample. Further research in the form of prospective registries, i.e. reporting systems in which cases of assisted suicide can be documented, and on details of procedural aspects may support transparency and standardization. Furthermore, qualitative analyses, for example in the form of semi-structured interviews with a sub-population of respondents or further professionals could increase the depth of data and contribute to further understanding.

Whether and how AS should be explicitly regulated by law is a subject of controversy in Germany [9–11, 40–42]. In particular, (renewed) criminal law regulation is often viewed critically [40–42]. However, procedural requirements could well be suitable for establishing a standardized procedure for assessing decisional capacity and providing information and counseling. This could include the assessment of decisional capacity by more than one person and the requirement that the assessment of decisional capacity/counseling and AS may not be carried out by the same person [40].

A reform of the “German Narcotics Act” to specify the use of pentobarbital for the purpose of AS in Germany would also be an option [41]. Furthermore, the aforementioned prospective registries for recording and making transparent AS practice also appear to be potential subjects for legal regulations and are intended as part of the planned “Suicide Prevention Act” in Germany [43]. A wide range of professional groups and laypeople receive requests for AS in Germany. Establishing standards of practice is of utmost importance in order to facilitate responsible practice in the future.

### Limitations

Our data is limited to a convenience sample of people who signed up for a webinar on AS. This group is, therefore, not representative and might be biased, since the participants may have been particularly interested in the topic for various reasons. In particular, our sample shows a marked gender imbalance, with 63% female and 35% male participants, which represents a possible source of bias and which might be due to a self-selection effect or reflect the gender distribution in the professional groups mostly represented in our study (physicians, psychologists, nurses). Furthermore, we did not collect data on requesters directly, but only self-reported data based on the reports of people who received the requests. The reliability of the data can therefore be questioned. In addition, there may be socially desirable response behavior. Due to the relatively small sample size, no statistical comparison was carried out between the group of requesters and the subpopulation that actually died by AS. The informative value of the comparison between the two groups is, therefore, limited. Furthermore, only what was written can be evaluated from the free-text responses. The fact that something was not written does not indicate with certainty that it was not actually the case. Due to the relative brevity and superficiality of the free-text responses, interpretation was difficult in some cases and the informative value is, therefore, limited.

## Supplementary Information

Supplementary Material 1.

## Data Availability

The datasets used analyzed during the current study are available from the corresponding author on reasonable request.
