# Supplementary material for: Assisted suicide in Germany: a survey on requests for and current practices of assisted suicide
Source: BMC Med Ethics. 2026 Feb 13;27:41. doi: 10.1186/s12910-026-01406-6 (PMC12930711; doi:10.1186/s12910-026-01406-6)
Supplement: Supplementary file 1 — Supplementary Material 1. [file 12910_2026_1406_MOESM1_ESM.pdf]

Assisted suicide in Germany: A survey on requests for and current practices of assisted suicide

Sommerlatte S.[1] , Droese E[1] ., Bausewein C.[2], Pollmächer T.[3] , Marckmann G.*[4] , Simon A.*[5] , Schildmann J.[1] *

*contributed equally

1. Institute for History and Ethics of Medicine, Interdisciplinary Center of Health Sciences, Martin Luther University of Halle-Wittenberg, Halle (Saale)
2. Department of Palliative Medicine, LMU University Hospital, Munich, Germany
3. Center for Mental Health, Ingolstadt Hospital, Ingolstadt; Department of Psychiatry and Psychotherapy, LMU University Hospital, Ludwig Maximilian University of Munich, Munich, Germany.
4. Institute of Ethics, History and Theory of Medicine, LMU Munich, Munich, Germany
5. Academy of Ethics in Medicine; Department of Medical Ethics and History of Medicine, University Medical Center Göttingen, Göttingen, Germany.

Corresponding author:

Dr. med. Sabine Sommerlatte, M. mel.

ORCID: https://orcid.org/0000-0001-6239-4349

sabine.sommerlatte@medizin.uni-halle.de

Additional file 1: Excerpt of the study questionnaire (author translation)

**Information about the respondents**	
What was your role in the case you reported? (Multiple answers possible)	☐ Psychologist/psychotherapist<br>☐ Social worker<br>☐ Nursing professional<br>☐ Pastoral caregiver<br>☐ Lawyer<br>☐ Physician<br>☐ Physical therapist/occupational therapist/speech therapist<br>☐ Volunteer (hospice association, visiting service, etc.)<br>☐ Relative<br>☐ Friend/acquaintance<br>☐ Other (please specify): … *Free text field …*
Age of the reporting person (years)	☐ Under 18<br>☐ 18-30<br>☐ 31-40<br>☐ 41-50<br>☐ 51-60<br>☐ 61-70<br>☐ 71-80<br>☐ 81-90<br>☐ 91-100<br>☐ >100
Gender of the reporting person	☐ Male<br>☐ Female<br>☐ Diverse
**Experience and personal attitude**	
Please indicate how often you have been asked for assistance with suicide in the last 12 months.	... *Free text field ...*
Please indicate how often you have assisted in suicide in the last 12 months.	... *Free text field ...*
**Information about the person you are reporting on**<br>Below, we ask you to provide information about the person who most recently requested assistance with suicide and/or died by assisted suicide. It is quite possible that you will not be able to provide information on all questions. In case of missing information or uncertainty, we ask you to indicate this in the mark the appropriate answer option.	
Gender of the person you are reporting on	☐ Male<br>☐ Female<br>☐ Diverse<br>☐ Unknown

Age of the person you are reporting on (years)	☐ Under 18<br>☐ 18-30<br>☐ 31-40<br>☐ 41-50<br>☐ 51-60<br>☐ 61-70<br>☐ 71-80<br>☐ 81-90<br>☐ 91-100<br>☐ >100<br>☐ Unknown
School leaving certificate	☐ Secondary school<br>☐ Intermediate school<br>☐ Grammar school<br>☐ No school-leaving certificate<br>☐ Unknown<br>☐ Other school-leaving certificate (please specify): … *Free text field …*
Training/education	☐ Apprenticeship<br>☐ University<br>☐ Unknown<br>☐ Other (please specify): … *Free text field …*
Social environment (multiple answers possible)	☐ Single<br>☐ Living in a partnership<br>☐ Married<br>☐ Child(ren)<br>☐ Widowed<br>☐ Unknown<br>☐ Further details (please specify): ... *Free text field ...*
Financial situation of the person you are reporting on	☐ The person concerned was able to live comfortably<br>☐ The person concerned was able to get by<br>☐ The person concerned had difficulty getting by<br>☐ The person concerned had great difficulty getting by<br>☐ Unknown
Illnesses (multiple answers possible)	☐ Cancer (please specify the diagnosis as precisely as possible): ... *Free text field ...*<br>☐ Neurological disease (please specify the diagnosis as precisely as possible): … *Free text field …*<br>☐ Cardiovascular disease (please specify the diagnosis as precisely as possible): … *Free text field …*<br>☐ Respiratory disease (please specify the diagnosis as precisely as possible): … *Free text field …*

	☐ Endocrine disorder (please specify the diagnosis as precisely as possible): … *Free text field …*<br>☐ Metabolic disease (please specify the diagnosis as precisely as possible): … *Free text field …*<br>☐ Gastrointestinal disease (please specify the diagnosis as precisely as possible): … *Free text field …*<br>☐ Infectious disease (please specify the diagnosis as precisely as possible): … *Free text field …*<br>☐ Musculoskeletal disease (please specify the diagnosis as precisely as possible): … *Free text field …*<br>☐ Mental/psychiatric disorder (please specify the diagnosis as precisely as possible): … *Free text field …*<br>☐ Other (please specify the diagnosis as precisely as possible): … *Free text field …*<br>☐ Unknown<br>☐ No illness
Was the person concerned cared for by a palliative care and/or hospice team?	☐ Yes<br>☐ No<br>☐ Not known
Would you have been surprised if the person you are reporting on had died of natural causes within 6 months of requesting assistance with suicide?	☐ Yes<br>☐ No<br>☐ Don't know
Where did the person live/stay at the time of the request to you?	☐ Private home<br>☐ Care facility<br>☐ Hospital<br>☐ Palliative care unit<br>☐ Hospice<br>☐ Unknown<br>☐ Other: … *Free text field …*
What happened after the request?	☐ Assistance with suicide was provided, and I can provide details<br>☐ Assistance with suicide was provided, but I cannot provide any further information on this (if<br>☐ you select this answer option, the survey will end as soon as you click "Continue")<br>☐ No action was taken<br>☐ I have no further information on the case (if you select this answer option, the survey will end as soon as you click "Continue")
Please state the year in which the assisted suicide took place.	... *Free text field ...*

How much time elapsed between the first request for assisted suicide and its implementation?	... *Free text field* ...
In your opinion, was the person concerned fully informed about their situation, the available options for action, and the consequences?	☐ Yes<br>☐ No<br>☐ Don't know<br>☐ Unknown
Who informed the person concerned about the options available?	... *Free text field* ...
Decisional capacity (Decisional capacity is generally understood to mean that the person concerned has understood the information relevant to the decision, including the consequences, and is able to make and communicate a decision in accordance with their preferences)	☐ The person concerned had the necessary decisional capacity with regard to assistance in suicide<br>☐ The person involved did NOT have the necessary decisional capacity with regard to assistance in suicide<br>☐ Don't know<br>☐ Unknown
To your knowledge, who assessed the decisional capacity and how?	☐ Unknown<br>☐ Known (please specify) ... *Free text field* ...
Voluntariness (in the sense of absence of pressure and undue influence by other persons)	☐ In the context of the (requested) assistance with suicide, the voluntariness of the actions was guaranteed<br>☐ In the context of (requested) assistance in suicide, the voluntariness of the actions was NOT guaranteed<br>☐ Not known
Where did the assisted suicide take place?	☐ Private area<br>☐ Care facility<br>☐ Hospital<br>☐ Palliative care unit<br>☐ Hospice<br>☐ Unknown<br>☐ Other (please specify): … *Free text field* …
To your knowledge, who was present? (Multiple answers possible)	☐ Relatives<br>☐ Friends/acquaintances<br>☐ Caregiver<br>☐ Physician<br>☐ Unknown<br>☐ Other persons (please specify): … *Free text field* …
How was the assisted suicide carried out? (e.g., what means, who procured it, dose, method of administration, and any other information you can provide)	☐ Unknown<br>☐ Please describe: … *Free text field* …
